# Neoadjuvant exemestane or exemestane plus docetaxel and cyclophosphamide tailored by clinicopathological response to 12 weeks' exemestane exposure in patients with estrogen receptor‐positive breast cancer: A multicenter, open‐label, phase II study

**DOI:** 10.1002/cam4.2423

**Published:** 2019-07-30

**Authors:** Nobuaki Sato, Norikazu Masuda, Takashi Morimoto, Takayuki Ueno, Chizuko Kanbayashi, Koji Kaneko, Hiroyuki Yasojima, Shigehira Saji, Hironobu Sasano, Satoshi Morita, Shinji Ohno, Masakazu Toi

**Affiliations:** ^1^ Department of Breast Oncology Niigata Cancer Center Hospital Niigata Japan; ^2^ Department of Surgery, Breast Oncology National Hospital Organization Osaka National Hospital Osaka Japan; ^3^ Department of Breast Surgery Yao Municipal Hospital Osaka Japan; ^4^ Breast Surgical Oncology, Breast Oncology Center Cancer Institute Hospital Tokyo Japan; ^5^ Department of Medical Oncology Fukushima Medical University Fukushima Japan; ^6^ Department of Pathology Tohoku University Miyagi Japan; ^7^ Department of Biomedical Statistics and Bioinformatics Kyoto University Graduate School of Medicine Kyoto Japan; ^8^ Breast Oncology Center Cancer Institute Hospital Tokyo Japan; ^9^ Department of Surgery (Breast Surgery), Graduate School of Medicine Kyoto University Kyoto Japan

**Keywords:** aromatase inhibitors, breast neoplasms, docetaxel and cyclophosphamide, Ki67 labeling index, tailored therapy

## Abstract

Our aim was to investigate the efficacy and safety of initial neoadjuvant endocrine therapy with exemestane alone followed by tailored treatment, either continued exemestane monotherapy or exemestane plus docetaxel–cyclophosphamide (TC) combination therapy, in postmenopausal patients with primary invasive estrogen receptor–positive, human epidermal growth factor receptor 2–negative, stage I‐IIIA breast cancer and Ki67 labeling index ≤30%. In this open‐label phase II study, patients initially received exemestane 25 mg/d for 12 weeks. Responders were defined as patients who achieved complete response (CR), partial response (PR) with Ki67 labeling index ≤5% after treatment, or stable disease with Ki67 labeling index ≤5% both before and after treatment. For the subsequent 12 weeks, exemestane monotherapy was continued for responders (group A), whereas nonresponders received exemestane plus four cycles of TC (docetaxel 75 mg/m^2^ and cyclophosphamide 600 mg/m^2^ every 3 weeks) (group B). Clinical response rate (ie the proportion of patients with CR or PR) at 24 weeks was the primary endpoint. Of 64 patients provisionally enrolled between December 2010 and May 2016, 58 (median age 60 years) started the study treatment. Five patients discontinued treatment in the initial exemestane monotherapy period, and 39 completed the study treatment. Clinical response rates at 8‐12 and 24 weeks were 71% (10/14, 95% confidence interval [CI] 41.9%‐91.6%) and 57% (8/14, 95% CI 28.9%‐82.3%), respectively, in group A, and 16% (4/25, 95% CI 4.5%‐36.1%) and 56% (14/25, 95% CI 34.9%‐75.6%), respectively, in group B. Grade ≥3 adverse events were reported in 8% (1/15) and 53% (20/38) in group A and group B, respectively. The tailored treatment maintained the favorable clinical response to exemestane alone in responders and improved clinical response in nonresponders.

**Trial number:**

UMIN000004752 (UMIN Clinical Trials Registry).

## Graphical abstract

Change in Ki67 labeling index over the course of the study in patients receiving continued exemestane monotherapy (A) and those receiving exemestane plus docetaxel–cyclophosphamide) (B).

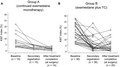

## 1 INTRODUCTION

Neoadjuvant treatment for breast cancer is commonly used to reduce tumor size, rendering a previously inoperable tumor fully resectable,^1^ or an operable tumor for which mastectomy had been indicated removable by breast‐conserving surgery (BCS).^2^ Over the past few decades, evidence has been accumulating to support the use of neoadjuvant endocrine therapy as an alternative option to neoadjuvant chemotherapy in postmenopausal women with estrogen receptor (ER)‐positive breast cancer.^3^ One key study, a 2016 meta‐analysis of data from 3490 patients with localized ER‐positive breast cancer, showed that neoadjuvant treatment with endocrine agents, either alone or in combination with other therapies, is as effective as neoadjuvant chemotherapy.^4^ The meta‐analysis also showed that third‐generation aromatase inhibitors (ie anastrozole, exemestane, and letrozole) are more effective than tamoxifen in this treatment setting. An additional finding, that neoadjuvant endocrine therapy is also associated with lower toxicity than neoadjuvant chemotherapy, is of particular interest to clinicians treating postmenopausal patients, because this group of patients tend to be older and, therefore, more likely to find the adverse effects of chemotherapeutic drugs intolerable or have difficulty attending chemotherapy appointments.^5^

Neoadjuvant therapy provides clinicians with the opportunity to assess tumor responsiveness to treatment. Research efforts have been focused on establishing an index for predicting benefit from and evaluating the effectiveness of neoadjuvant endocrine therapy in patients with ER‐positive breast cancer.^6^ Such an index could be used to identify patients most likely to benefit from neoadjuvant endocrine therapy and guide subsequent clinical decision making, for example by enabling physicians to tailor neoadjuvant therapy to maximize its effectiveness in individual patients. Thus, patients with an inadequate response to neoadjuvant endocrine monotherapy could be switched to another treatment, for example a combination of endocrine therapy and chemotherapy, or immediate surgery.

We have investigated the use of the Ki67 labeling index, which represents the percentage of Ki67‐positive carcinoma cells, for monitoring response to neoadjuvant endocrine therapy and guiding subsequent treatment. Specifically, we have focused on changes in Ki67 labeling index in response to exposure to exemestane,^7^ and the interpretation of such changes to tailor neoadjuvant therapy to maximize its therapeutic effects.^8^

On‐treatment Ki67 threshold values for switching from neoadjuvant aromatase inhibitor therapy to neoadjuvant chemotherapy have been established by the study of preoperative letrozole^9^ and the IMPACT trial.^10^ In the former, Ki67 >10% at 1 month was associated with higher preoperative endocrine prognostic index (PEPI) scores derived from the pT stage, pN stage, Ki67 labeling index level, and ER status of the surgical specimen (*P *= .01); a small number of patients in the PEPI‐0 group (*P *= .08); and worse relapse‐free survival rate (*P *= .0016). Similarly, in the IMPACT trial, a 2‐week Ki67 >10% predicted higher PEPI score (*P *= .001), a small number of patients in the PEPI‐0 group (*P *= .004), and worse relapse‐free survival rate (*P *= .008). Combining the results of these studies, there was only one PEPI‐0 case among 51 patients with a 2‐ to 4‐week Ki67 value >10%. Therefore, according to the PEPI model, patients with a Ki67 value of 10% at 2‐4 weeks had a <2% chance of a favorable PEPI score, which would allow them to safely avoid chemotherapy under current guidelines.

We recently reported the results of a multicenter, open‐label, phase II study in which Ki67 labeling index before and after an initial period of endocrine therapy, namely, treatment with exemestane alone (25 mg/d, administered orally), was used to help classify postmenopausal patients with ER‐positive breast cancer as responders or nonresponders.^8^ Responders (ie patients with adequate biologic response, ie change in Ki67 index, as well as clinical response) continued to receive exemestane monotherapy, whereas nonresponders (again based on biologic as well as clinical response) were switched to chemoendocrine therapy, namely, exemestane plus low‐dose cyclophosphamide (50 mg/d, administered orally). The results provided support for the potential benefit of this tailored approach to the neoadjuvant treatment of ER‐positive breast cancer, because clinical response rate (ie the proportion of patients with complete response [CR], or partial response [PR]) remained high in responders and improved in nonresponders. Thus, the results showed that therapeutic effects could be maximized while minimizing the incidence of adverse events (AEs) associated with chemotherapeutic drugs.

The present study was conducted in parallel with our previously reported study. We used a similar study design to investigate the efficacy and safety of initial neoadjuvant endocrine therapy with exemestane alone followed by tailored treatment: continued exemestane monotherapy for responders or exemestane plus docetaxel–cyclophosphamide (administered intravenously, ie TC) combination therapy for nonresponders. The hypothesis was that in nonresponders to the initial exemestane monotherapy, the TC regimen might have different therapeutic effects than the low‐dose cyclophosphamide, administered orally, used in our previous study,^8^ with acceptable tolerability. We selected the TC regimen as chemotherapy because docetaxel and cyclophosphamide have been shown to be equally effective for hormone receptor–positive disease as well as receptor–negative disease.^11^ Moreover, a regimen of four cycles of TC has been shown to be superior to the standard doxorubicin–cyclophosphamide combination therapy and is tolerable for both older and younger patients.^11^

The TC regimen was chosen rather than anthracycline and cyclophosphamide (administered intravenously, ie AC). TC is less likely than AC to cause bone marrow toxicity (anthracyclines cause bone marrow toxicity and increase the risk of leukemia or myelodysplasia due to bone marrow damage, and some cases have been fatal).^12^ Although TC is associated with higher rates of febrile neutropenia, it has better tolerability than AC, regardless of patient age. Furthermore, anthracycline causes cardiac toxicity.^13^ Older women who received anthracycline‐based adjuvant chemotherapy have much higher rates of chronic heart failure years after completion of treatment, suggesting that the long‐term toxicity of current anthracycline‐containing regimens may be underestimated.^14^

Additionally, we examined the clinical usefulness of Ki67 labeling index as a marker.

## 2 PATIENTS AND METHODS

### 2.1 Study design and patients

**Figure 1:**
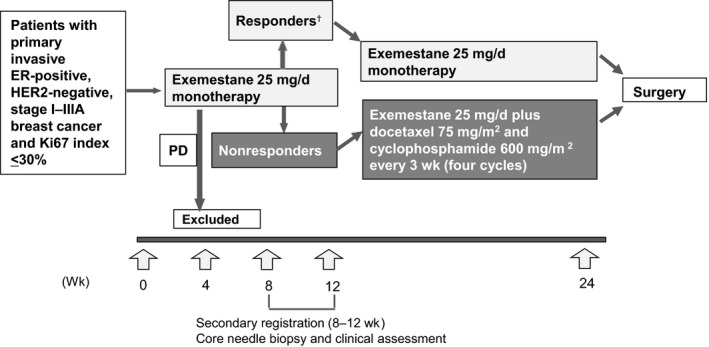
Study design. ^†^Responders were defined as patients with complete response, partial response with Ki67 labeling index <5% after treatment, or stable disease with Ki67 labeling index <5% before and after treatment. PD, progressive disease

The study design, which closely followed the one used in our previously reported study,^8^ is shown in Figure 1. Both are multicenter, open‐label, phase II studies. The key difference is that, in this study, patients allocated to the combination therapy group received four cycles of docetaxel and cyclophosphamide (administered intravenously) rather than low‐dose cyclophosphamide (administered orally). Patients were registered from eight institutions across Japan using a central registration method. The eligibility criteria were postmenopausal status; diagnosis of primary invasive ER‐positive, human epidermal growth factor receptor 2 (HER2)–negative, stage I‐IIIA (T1c–T3, N0‐2, M0) breast cancer (confirmed by needle biopsy or histological findings); Ki67 labeling index ≤30%; Eastern Cooperative Oncology Group performance status 0 or 1; indication for partial or total mastectomy; and no previous treatment for cancer. ER‐positive status was confirmed by immunohistochemistry, and HER2‐negative status by either immunohistochemistry or fluorescence in situ hybridization, as described previously.^8^ Patients with or without axillary lymph node metastasis were eligible for inclusion. Patients with lobular or mucinous cancer were excluded.

Hematological, cardiac, hepatic, and renal function were confirmed as adequate in all patients by the results of laboratory tests. Each patient's attending physician, having considered the various treatment options, had judged neoadjuvant endocrine therapy to be appropriate in each case.

The study complied with the Declaration of Helsinki (1975, as revised in 2008) and the Ethical Guidelines for Clinical Research of the Ministry of Health, Labour and Welfare of Japan. The institutional review board of each participating institution reviewed and approved the study protocol. All patients provided written informed consent.

The study has been registered with the University Hospital Medical Information Network Clinical Trials Registry (http://www.umin.ac.jp/ctr/index-j.htm); its unique trial number is UMIN000004752. The Japan Breast Cancer Research Group trial number is JBCRG‐11TC.

### 2.2 Study treatment

In the initial treatment period, all patients received endocrine therapy: exemestane 25 mg/d administered orally for 12 weeks. Secondary registration took place at 8‐12 weeks; during this time, the response, including effects on Ki67 labeling index values, was assessed by core needle biopsy and clinical assessment. Based on the results of this assessment, patients were assigned to either continued endocrine therapy or chemoendocrine therapy for the subsequent 12 weeks, specifically continued exemestane monotherapy for responders (group A) or exemestane plus four cycles of TC (docetaxel 75 mg/m^2^ and cyclophosphamide 600 mg/m^2^ every 3 weeks) for nonresponders (group B). The TC regimen was administered by intravenous infusion on day 1 in each cycle, as in a previous study.^15^

Responders were defined as patients with CR, those with PR and Ki67 labeling index ≤5% after treatment, and those with stable disease (SD) and Ki67 labeling index ≤5% both before and after treatment, and nonresponders as patients with PR and Ki67 labeling index >5% after treatment, and those with SD and Ki67 labeling index >5% either before or after treatment. In cases in which Ki67 labeling index could not be determined, patients with CR or PR were classified as responders, whereas patients with SD were classified as nonresponders. One concern was that it might not be possible to determine Ki67 labeling index values in patients who missed the core needle biopsy procedure in the on‐treatment setting. Therefore, in cases in which Ki67 labeling index values were unavailable, we considered assessment by more objective measurements (ie ultrasound, computed tomography [CT], or magnetic resonance imaging [MRI]), in addition to caliper measurement, sufficient to classify patients with CR or PR as responders.

Progressive disease (PD) was considered to indicate inadequate efficacy of exemestane monotherapy in the initial treatment period. Therefore, the study treatment was discontinued for patients with PD.

Details of the concomitant and post‐study therapy are available in our previous report.^8^

### 2.3 End points

Clinical response rate, defined as the proportion of patients with either CR or PR, at 24 weeks was the primary end point. Secondary end points included pathological response; changes in tumor size (maximum diameter at 8‐12 and 24 weeks vs maximum diameter at baseline); change in Ki67 labeling index and PEPI scores derived from the pT stage, pN stage, Ki67 labeling index level, and ER status of the surgical specimen; clinical benefit, assessed as increased BCS rate (ie increased proportion of patients undergoing BCS); and incidence of AEs. AEs were defined as new or worsening of subjective or objective symptoms in a patient who received the study treatment, or an abnormal change in laboratory test values, that does not necessarily have a causal relationship with the study drug.

### 2.4 Efficacy assessment

Efficacy was evaluated using the Response Evaluation Criteria in Solid Tumors, version 1.1. At baseline and at 8‐12 and 24 weeks, tumors were assessed by visual inspection and palpation of the breast. Additionally, at the discretion of the attending physician, ultrasound, CT, or MRI were performed to determine tumor size, although it was specified that CT or MRI had to be performed in addition to ultrasound for tumors with maximum diameter >4 cm. If multiple tumors were present, up to five were selected for measurement.

Clinical response was determined by comparing the maximum diameter of tumor(s) with corresponding baseline measurements, or by noting the development of new lesions. The sum of percentages of patients with CR or PR was used as the clinical response rate.

The pathology committee determined pathological response (see section 2.5). It was categorized using the modified criteria described by Miller et al^16^: pathological CR (pCR) was tentatively defined in the present study when no residual carcinoma cells were detected at the original site of the tumor during careful histopathological evaluation of surgical specimens; pathological PR (pPR), when a decrease in cellularity of carcinoma cells and a concomitant increase in fibrosis or stromal hyalinization were detected at the site of the primary tumor during careful histopathological evaluation of surgical specimens; or no response, when no histological changes were identified in carcinoma cells in surgical specimens.

### 2.5 Pathological assessment

As in our previously reported studies,^7^, ^8^ the possibility of interlaboratory variability was eliminated by use of a single laboratory for immunohistochemical staining in Ki67 labeling index measurement. Details of how tissue specimens were obtained and fixed, and how tissue sections were prepared, are as described previously.^8^ For each patient, an unstained slide was sent from each study site to a central pathology laboratory (Department of Pathology, Tohoku University School of Medicine) for immunohistochemical staining and evaluation of ER, progesterone receptor, HER2, and Ki67.

For the purposes of diagnosis, the pathology committee examined tissue samples obtained before the start of the study treatment. Pathological response after the initial exemestane monotherapy and at the completion of the study treatment was determined by examination of tissue samples collected at 8‐12 weeks and surgical specimens, respectively. The committee also used tissue samples collected at 8‐12 weeks for interim assessment of Ki67 labeling index, and surgical specimens for final assessment of Ki67 labeling index.

Low and high tumor cell proliferation were tentatively defined as Ki67 labeling index ≤5% and Ki67 labeling index >5%, respectively, in the present study. In a previous study, in which patients received 24 weeks of exemestane monotherapy, no patients with Ki67 labeling index <15% at baseline had PD, and median Ki67 labeling index decreased from 10 (range, 0‐55) to 2 (range, 0‐34), that is, ≤5%, in patients who achieved pPR.^7^ Based on this result, Ki67 labeling index ≤5% and favorable clinical response to the initial treatment were chosen as the criteria for continuation of exemestane monotherapy.

### 2.6 Safety assessment

Adverse events were recorded every 4 weeks throughout the 24‐week treatment period. Grading of AEs was done in accordance with the National Cancer Institute Common Terminology Criteria for AEs, version 4.0 (Japanese Clinical Oncology Group edition).^17^

### 2.7 Follow‐up

Overall survival and relapse‐free survival have been specified in the protocol as secondary end points. Ongoing follow‐up of responders and nonresponders will enable us to report, in due course, the effects on long‐term survival of the tailored approach to neoadjuvant treatment of ER‐positive breast cancer described in this article. Further results will be published when sufficient data become available.

### 2.8 Statistical analyses

The target sample size was 60 patients. The rationale for this target sample size is explained in the report of our previously reported study of tailored neoadjuvant endocrine and chemoendocrine therapy.^8^

Summary statistics were used to evaluate tumor response. Clinical response rates, with 95% confidence intervals, were calculated. Continuous variables were compared using the Mann‐Whitney *U* test. Clinical response rates at 8‐12 and 24 weeks were compared using McNemar's test. The incidence of different AEs, stratified by severity (grades 1‐4), was calculated.

An intent‐to‐treat analysis was used to evaluate the efficacy of the tailored treatment; data from all the eligible patients were used. The full analysis set was defined as data from all patients who had completed the initial period of treatment with exemestane alone and who started subsequent therapy with either continued exemestane monotherapy or exemestane plus TC. The safety analysis was carried out using data from all patients who received at least one dose of exemestane.

Univariate and multivariate analyses were carried out, using data from the full analysis set, to identify factors associated with response or nonresponse to the initial treatment. Fisher's exact test was used to compare PEPI scores in groups A and B.

IBM SPSS Statistics 23.0 (IBM Corp.) and R version 3.2.2 (R core team, R Foundation for Statistical Computing) were used for all statistical analyses.

## 3 RESULTS

### 3.1 Patients

**Figure 2:**
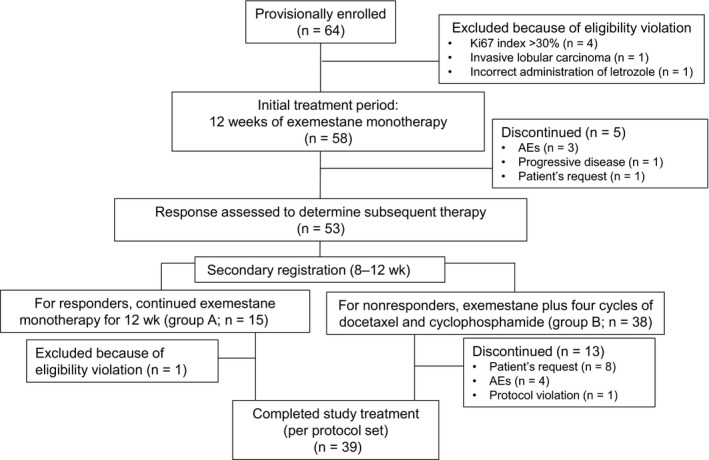
Patient disposition during the study. AE, adverse event

Figure 2 shows the progress of patients through the phases of the study. Of 64 patients provisionally enrolled between December 2010 and May 2016, six were excluded because of violations of the eligibility criteria. Therefore, 58 patients were eligible for the study and started the initial 12‐week period of treatment with exemestane alone. Their data were used for the intent‐to‐treat set.

The baseline characteristics of the 58 eligible patients are summarized in Table 1. All had ER‐positive, HER2‐negative breast cancer. Until the start of the study, none had received treatment for breast cancer. Ki67 labeling index values were available for all patients.

**Table 1:** Baseline patient characteristics (n = 58)^a^

Characteristic	n (%)^b^
Age, years (median and range)	60 (53‐67)
Tumor stage	
T1	10 (17)
T2	46 (79)
T3	2 (3)
Nodal status	
N0	49 (84)
N1	9 (15)
Clinical stage	
I	9 (15)
IIA	41 (71)
IIB	6 (10)
IIIA	2 (3)
Maximum diameter of tumor, mm (median and range)	
Caliper measurement	25 (10‐70)
Ultrasound measurement	23 (12‐42)
CT/MRI	22 (12‐71)
Estrogen receptor status	
Positive	58 (100)
Negative	0
HER2 status	
Positive	58 (100)
Negative	0
Progesterone receptor status	
Positive	51 (88)
Negative	7 (12)
Ki67 labeling index	11.9 (5.0‐30.0)

Abbreviation: HER2, human epidermal growth factor receptor 2.

a Intent‐to‐treat set. For patients with multiple tumors, the data are for representative lesions only.

b Unless otherwise indicated.

Five patients discontinued treatment during the initial exemestane monotherapy period. Therefore, at 8‐12 weeks the response to the initial treatment was assessed in 53 patients (the full analysis set comprised data from these patients). Of these, 15 were classified as responders (PR and Ki67 labeling index ≤5% after treatment, eight patients; SD and Ki67 labeling index ≤5% both before and after treatment, seven patients), and 38 were classified as nonresponders (PR and Ki67 labeling index >5% after treatment, five patients; SD and Ki67 labeling index >5% either before or after treatment, 33 patients). In group A, one patient was excluded because of violation of the eligibility criteria before they began their second 12‐week period of exemestane monotherapy. In group B, 13 patients discontinued treatment. Therefore, 39 patients completed the study (the per protocol set comprised data from these patients).

The proportion of patients in the per‐protocol set who complied with treatment, with or without dose reduction, was 100% (14/14) in group A and 80% (20/25) in group B.

### 3.2 **Clinical response rates at 8**‐**12 and 24 weeks**

Table 2 shows the clinical response rates (ie sums of the percentages of patients with CR or PR) at 8‐12 and 24 weeks in groups A and B. Clinical response rate at 8‐12 weeks was higher in group A than in group B (71% vs 16%, *P *= .001). In patients in group A, that is, those who responded to the initial treatment, clinical response rate remained high with continued exemestane monotherapy (71% at 8‐12 weeks, 57% at 24 weeks). In patients in group B, that is, those with an inadequate response to the initial treatment, clinical response rate improved significantly with subsequent treatment with exemestane plus TC (16% at 8‐12 weeks, 56% at 24 weeks; *P *= .02).

**Table 2:** Changes in clinical response rate over the course of the study^a^

Time (weeks)	Group A (continued exemestane monotherapy)		Group B (exemestane plus TC)	
	n (%)	95% CI (%)	n (%)	95% CI (%)
8‐12	10/14 (71)^b^	41.9‐91.6	4/25 (16)	4.5‐36.1
24	8/14 (57)	28.9‐82.3	14/25 (56)^c^	34.9‐75.6

Abbreviations: CI, confidence interval; TC, docetaxel and cyclophosphamide (four cycles).

a Clinical response rate defined as the sum of the percentages of patients with complete response or partial response.

b Clinical response rate at weeks 8‐12 was higher in group A than in group B (*P *= .001, McNemar's test).

c In group B, clinical response rate improved significantly with subsequent treatment with exemestane plus TC (*P *= .02, McNemar's test).

Regarding the primary end point, the clinical response rate at 24 weeks was almost the same in group A as in group B (57% and 56%, respectively).

### 3.3 Change in tumor size

**Figure 3:**
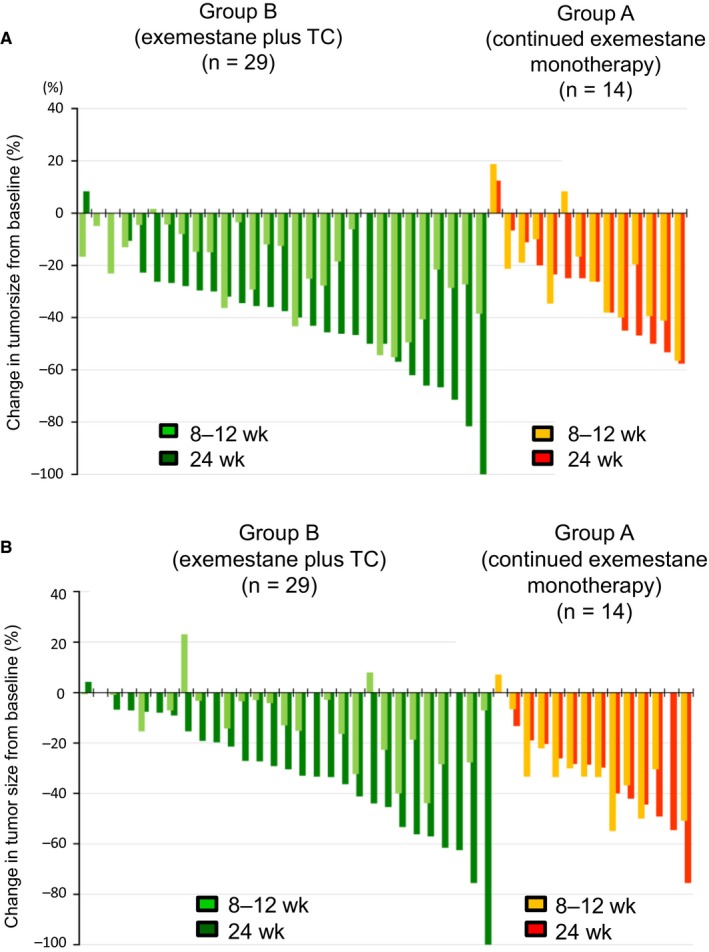
Waterfall plots showing clinical response to exemestane‐based neoadjuvant therapy at 8‐12 wk and 24 wk in patients who responded to initial treatment with exemestane alone and who continued to receive monotherapy (group A), and nonresponders, who were switched to exemestane plus docetaxel–cyclophosphamide (group B). Results obtained by (A) ultrasound and (B) computed tomography or magnetic resonance imaging. The horizontal axes indicate paired data from individual patients for whom data were available. The vertical axes show percentage change in tumor size from baseline; positive values indicate tumor progression, and negative values indicate tumor regression

Changes in tumor size from baseline, as measured in individual patients by ultrasound and CT or MRI at 8‐12 and 24 weeks, are shown in Figure 3. The results obtained with the different imaging modalities showed that in the clear majority of group B patients for whom tumor shrinkage was recorded at 8‐12 weeks, a greater degree of reduction in tumor size from baseline was recorded at 24 weeks. In contrast, the equivalent finding was recorded for about half of group A patients for whom data were available.

### 3.4 Change in Ki67 labeling index

**Figure 4:**
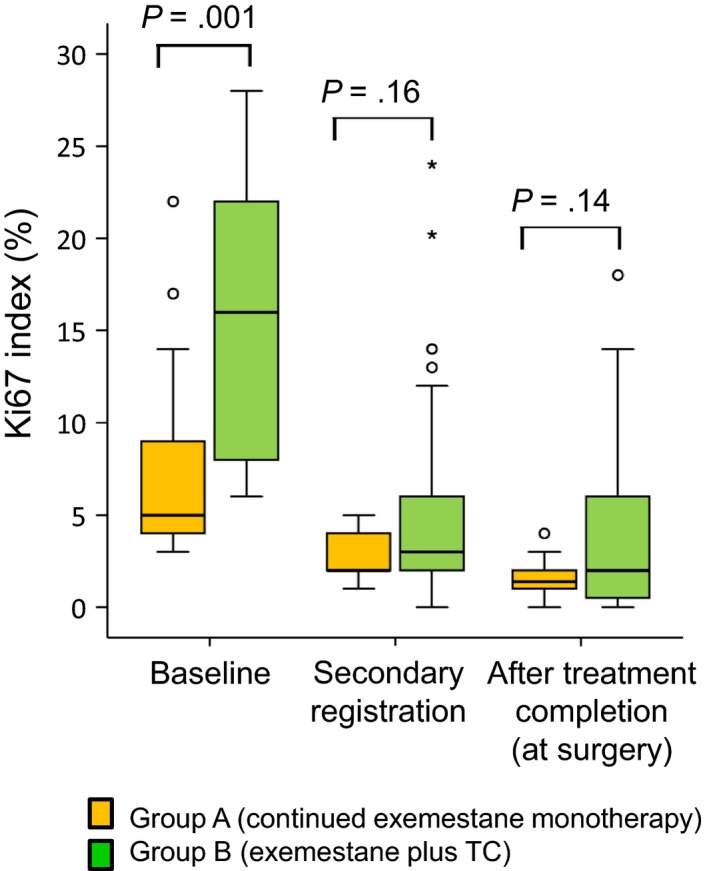
Change in median Ki67 labeling index in patients who responded to initial treatment with exemestane alone and who continued to receive exemestane monotherapy (group A), and nonresponders, who were switched to combination therapy with exemestane plus docetaxel–cyclophosphamide (group B). Data from the full analysis set. *indicates extreme outliers

Changes in median Ki67 labeling index are summarized in Figure 4. At baseline, median Ki67 labeling index was significantly lower in responders to the initial treatment with exemestane alone than in nonresponders (5.0% and 16.0%, *P *= .001). There were no significant differences between the two groups in median Ki67 labeling index at 8‐12 weeks (2.0% and 3.0%, respectively) or 24 weeks (1.4% and 2.0%, respectively).

**Figure 5:**
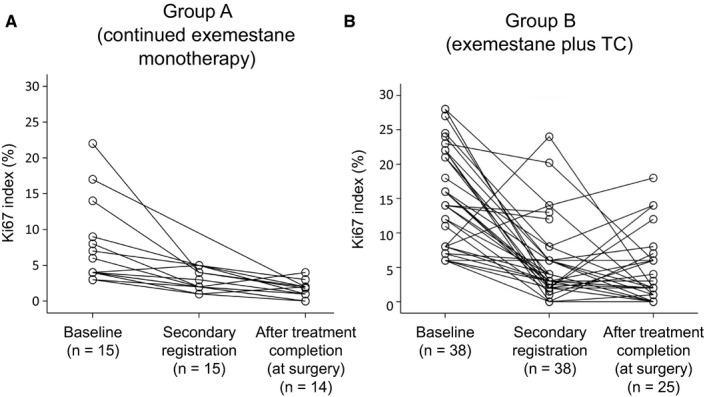
Change in Ki67 labeling index over the course of the study in individual patients in (A) group A (continued exemestane monotherapy) and (B) group B (exemestane plus docetaxel–cyclophosphamide). Baseline data from the full analysis set

Specific patterns of change in Ki67 labeling index in individual patients over time are shown in Figure 5. Of the patients who responded to the initial treatment with exemestane monotherapy, Ki67 labeling index decreased during this period in all except 1 patient, in whom Ki67 labeling index showed a minimal increase. In 1 patient, the decrease was substantial (about 28%). Ki67 labeling index either increased or decreased during the subsequent continued exemestane monotherapy up until surgery but remained <10% in all except 1 patient. Of the nonresponders to exemestane monotherapy, Ki67 labeling index decreased during the initial treatment period in all except 2 patients. At the end of exemestane monotherapy, 4 patients had Ki67 labeling index >10%. Ki67 labeling index decreased further in 3 of these patients during the subsequent exemestane plus TC therapy up until surgery. In 4 patients, Ki67 labeling index increased up to >10% during combination therapy, and in 3 of these patients the increase was substantial (about 20%‐40%).

### 3.5 Factors associated with response or nonresponse to the initial therapy

Table 3 summarizes the results of univariate and multivariate logistic analyses carried out to identify factors associated with classification of patients into responders or nonresponders, based on clinical response and change in Ki67 labeling index values in response to the initial therapy.

**Table 3:** Factors associated with response or nonresponse to the initial therapy (exemestane alone)

A. Univariate logistic analysis					
Factor	*B*	SE	OR^a^	*P*	95% CI
Age	−0.16	0.10	0.85	.08	0.69‐1.02
≥T2 (ref: T1)	0.76	0.74	2.14	.29	0.51‐8.61
N1 (ref: N0)	2.23	1.56	9.34	.04*	1.05‐1234.86
Histological grade ≥2 (ref: grade 1)	1.07	0.64	2.92	.08	0.87‐10.64
ER TS (ref: ≤7)	−1.56	0.95	0.21	.05	0.02‐1.02
PgR positive (ref: negative)	0.43	0.92	1.53	.62	0.24‐7.93
HER2 2+ (ref: negative)	0.44	0.72	1.55	.52	0.42‐6.96
Ki67 labeling index at baseline	0.17	0.06	1.19	.001*	1.07‐1.38
Ki67 labeling index at baseline ≥14 (ref: <14)	1.71	0.71	5.55	.008*	1.54‐24.88

B. Multivariate logistic analysis					
Factor	*B*	SE	OR^a^	*P*	95% CI
Starting model					
Age	−0.11	0.11	0.90	.33	0.71‐1.11
≥T2 (ref: T1)	0.33	0.79	1.39	.66	0.31‐5.88
N1 (ref: N0)	1.67	1.46	5.30	.19	0.52‐720.0
Histological grade ≥2 (ref: grade 1)	−0.19	0.99	0.83	.85	0.09‐5.05
ER TS (ref: ≤7)	−0.88	0.97	0.41	.33	0.04‐2.27
PgR positive (ref: negative)	0.29	1.06	1.33	.77	0.17‐10.02
HER2 2+ (ref: negative)	0.50	0.81	1.64	.52	0.37‐8.47
Ki67 labeling index ≥14 (ref: <14)	1.09	0.97	2.96	.24	0.48‐23.45
Best‐fit model					
N1 (ref: N0)	1.90	1.60	6.68	.12	0.68‐901.4
Ki67 labeling index ≥ 14 (ref: <14)	1.51	0.71	4.54	.02*	1.21‐20.76

Abbreviations: CI, confidence interval; ER, estrogen receptor; HER2, human epidermal growth factor receptor 2; OR, odds ratio; PgR, progesterone receptor; ref, reference category; SE, standard error.

a Odds ratios for nonresponse.

* *P *< .05.

In the univariate analysis, a significant association with likelihood of nonresponse to the initial period of treatment with exemestane alone was found for stage (N), a categorical variable, and Ki67 labeling index at baseline (a continuous variable) (Table 3A). Compared with patients with N0, patients with N1 were more likely to be switched to exemestane plus TC combination therapy (odds ratio, 9.34). Patients with higher pretreatment Ki67 labeling index at baseline were also more likely to be switched to exemestane plus TC (odds ratio, 1.19).

Both the univariate and the multivariate analysis showed that, compared with patients with Ki67 labeling index <14, patients with baseline Ki67 labeling index ≥14 were more likely to be switched to exemestane plus TC (odds ratios, 5.55 and 4.54, respectively; Tables 3A and B, respectively).

### 3.6 **Change in **PEPI

When the patients were tentatively stratified according to PEPI score (Table 4), no significant differences were detected between group A and group B (Fisher's exact test).

**Table 4:** Comparison of preoperative endocrine prognostic index (PEPI) scores in patients in group A and group B

PEPI score	Group A (continued exemestane monotherapy), n (%)	Group B (exemestane plus TC), n (%)
0	9/14 (64)	9/25 (36)
1‐3	4/14 (29)	10/25 (40)
≥4	1/14 (7)	6/25 (24)

Abbreviation: TC, docetaxel and cyclophosphamide (four cycles).

### 3.7 Pathological response

In group A, none of the patients had pCR, 10 had pPR, and there were 4 nonresponders. In group B, no patients had pCR, 30 had pPR, and 2 were nonresponders. The proportion of patients with pathological response was significantly higher in group B than in group A (*P* < .05, Fishers' exact test) (Table 5).

**Table 5:** Pathological response

Pathological response	Group A (continued exemestane monotherapy), n (%)	Group B (exemestane plus TC), n (%)	Both groups, n (%)	*P* (group A vs group B)
Pathological complete response	0	0	0 (0)	1.000
Pathological partial response	10 (71.4)	30 (78.9)^a^	40 (77.0)	.712
No response	4 (28.6)	2 (5.3)	6 (11.5)	.038
Not evaluable	0 (0)	6 (15.8)	6 (11.5)	.174
Total	14 (100)	38 (100)	52 (100)	

Abbreviation: TC, docetaxel and cyclophosphamide (four cycles).

a The proportion of patients with pathological response was significantly higher in group B than group A (*P* < .05, Fishers' exact test).

### 3.8 BCS rate

There was a significant difference in the proportion of patients who underwent BCS between groups A and B (93% and 56%, respectively; *P *= .009, Fisher's exact test).

### 3.9 Adverse events

Adverse events grade ≥3 were reported in 40% (21/53) of patients (group A, 8%, 1/15; group B, 53%, 20/38). The most common were leukopenia (37%, 14/38), neutropenia (32%, 12/38), and febrile neutropenia (16%, 6/38) during chemotherapy (group B).

## 4 DISCUSSION

The results of our present study, along with those of our most recently reported previous study,^8^ confirm the efficacy and safety of tailored neoadjuvant exemestane‐based endocrine and chemoendocrine therapy in postmenopausal patients with ER‐positive breast cancer and Ki67 labeling index ≤30%, using an approach based on both biologic and clinical criteria. Patients classified as responders to 12 weeks of neoadjuvant therapy with exemestane alone continued to benefit from its continuation for the subsequent 12 weeks. Patients who had an inadequate response to exemestane monotherapy benefited from a switch to neoadjuvant therapy with exemestane in combination with four cycles of TC. Thus, the favourable clinical response to exemestane alone was maintained in responders and the switch to combination therapy enabled clinical response to be achieved in nonresponders, to the extent that the proportion of patients with either CR or PR was almost the same in both groups by the end of the study treatment. Regarding safety, compared with exemestane monotherapy, exemestane plus TC combination therapy was associated with higher incidence of hematological AEs, but these were manageable.

Regarding reduction in tumor size from baseline, the second 12‐week treatment period was more likely to affect further shrinkage in nonreponders than in responders. This finding was presumably due to the enhancement of antiproliferative effect provided by the addition of chemotherapeutic drugs to the neoadjuvant therapy these patients received in the second treatment period. In contrast, for responders the effect on tumor volume of extended exemestane monotherapy was less predictable. A possible explanation is that the antiproliferative effect of inhibition of estrogen production by exemestane reached a plateau in some patients at 12 weeks, and that in these individuals continued exemestane exposure in the weeks thereafter conferred little additional benefit.

Ki67 is a nuclear antigen expressed during the growth and synthesis phases, but not the resting phase, of the cell cycle. It is therefore a marker of proliferation and has been investigated for its prognostic value in various cancers including gastrointestinal cancer,^18^ prostate cancer,^19^ and breast cancer.^20^, ^21^, ^22^

Ki67 labeling index, determined by immunohistochemical assessment of surgical pathology specimens, could be the most useful and practical laboratory parameter in the clinical management of breast cancer patients. Its clinical validity is reasonably well established.^6^ It has been shown to differentiate groups of patients according to outcome. Meta‐analyses of data from patients with early breast cancer have shown that higher levels of Ki67 labeling are associated with worse prognosis (data from 12 155 patients),^23^ including significantly shorter overall and disease‐free survival (data from 15 790 patients).^24^ These findings are also consistent with those of a more recent large‐scale retrospective study.^21^ Additionally, Ki67 labeling index has been shown to identify patients whose tumors are likely to respond to endocrine therapy, for example in the context of evaluation of response to both neoadjuvant chemotherapy^25^ and endocrine therapy.^7^, ^8^

An on‐treatment Ki67 threshold for switching from neoadjuvant aromatase inhibitor therapy to neoadjuvant chemotherapy has been established, using data from a study of preoperative letrozole.^9^, ^10^ We also consider tumor volume when determining the responder or nonresponder status of patients, using measurements obtained not only by caliper measurement but also by more objective measurements (ie ultrasound, CT, or MRI).

The potentially great clinical value of Ki67 labeling index as both a prognostic and a predictive biomarker is, however, hindered by various factors. These include problems establishing its analytical validity, such the lack of standardization of immunohistochemical assessment of Ki67 labeling index and the difficulty in assigning cutpoints for high versus low Ki67 labeling index.^26^, ^27^, ^28^ Despite these caveats, the use of Ki67 labeling index values after an initial period of neoadjuvant treatment with an aromatase inhibitor to triage postmenopausal patients with ER‐positive breast cancer to continued endocrine therapy, chemotherapy, or immediate surgery has been useful approach in clinical settings,^29^ and some authors consider a degree of variability in Ki67 labeling to be acceptable.^6^

The present study is part of our continuing investigation of the utility of Ki67 labeling index values for guiding neoadjuvant treatment strategy for individual postmenopausal patients with ER‐positive breast cancer. By determining the likelihood of achieving clinical response in each case, we aim to avoid the unnecessary use of chemotherapy in low‐risk patients. As in our previously reported study of tailored exemestane‐based neoadjuvant therapy, in which the same eligibility criteria were applied,^8^ patients were classified as responders or nonresponders primarily on the basis of Ki67 labeling index (using 5% as a cutpoint) and secondarily on the basis of clinical response. Thus, in both studies, responders included patients with SD, provided they also had Ki67 labeling index ≤5% both before and after treatment, and nonresponders included patients with PR, provided they also had Ki67 labeling index >5% after treatment.

The results of our present study, which are consistent with those of our previous study,^8^ demonstrate the antiproliferative effects of an initial 12 weeks of exemestane monotherapy and of subsequent exemestane therapy with or without chemotherapy. In both studies, although median Ki67 labeling index at baseline was significantly lower in patients later assessed as having responded to the initial period of exemestane monotherapy, no differences between responders and nonresponders were detected when response to the initial treatment period was assessed at 8‐12 weeks or at completion of the study treatment.

Consistent with the results of our previous study,^8^ substantial decreases in Ki67 labeling index were found in individual patients in the first 12 weeks of treatment, suggesting that tumors may be most susceptible to the antiproliferative effects of exemestane during this period. Other aromatase inhibitors, for example letrozole,^16^ have produced similar decreases in Ki67 labeling index.

The addition of docetaxel to the chemoendocrine therapy used in the present study may have enhanced its efficacy, thus explaining differences from our previous study in terms of the pattern of change in Ki67 labeling index in individual patients. In nonresponders in the present study, who were switched to exemestane plus TC, the range of Ki67 labeling index values decreased. In nonresponders in the previously reported study, who were switched to exemestane plus cyclophosphamide only, the opposite occurred.^8^

The results of our present study confirm that Ki67 labeling index values are sufficiently informative for use in triaging postmenopausal patients with ER‐positive breast cancer to continued exemestane monotherapy or exemestane plus TC. Compared with nodal status, a clinical characteristic closely correlated with tumor volume,^30^, ^31^, ^32^ Ki67 labeling index, which is a biologic characteristic, more reliably predicted response to initial exemestane monotherapy. In both our present and previously reported studies,^8^ patients with low cell proliferation in their carcinoma cells were more likely to respond to exemestane alone.

Higher Ki67 labeling index at baseline could therefore indicate that carcinoma cells were more resistant to treatment, and therefore justify a more aggressive first‐line neoadjuvant therapy. Thus, the use of chemotherapy or chemoendocrine therapy would be favoured over endocrine therapy, because the potential clinical benefits would outweigh the risk of AEs associated with the use of chemotherapeutic drugs.

A potential clinical benefit of neoadjuvant therapy is improved operability. In cases of breast cancer, the use of neoadjuvant therapy can enable tumors for which mastectomy had been indicated to be removed by BCS instead. This has been shown by the results of previous studies of neoadjuvant therapy with aromatase inhibitors in postmenopausal breast cancer patients.^4^, ^7^ In the present study, switching nonresponders from exemestane monotherapy to exemestane‐based chemotherapy did not increase the rate of conversion from mastectomy to BCS. On completion of the study treatment, only 56% of nonresponders underwent BCS, compared with a much higher proportion (93%) of responders to exemestane monotherapy. This finding contrasts with the results of our most recently reported previous study, in which about 70% of patients in each group underwent BCS.^8^

Differences in baseline characteristics of the study populations, including disease severity, may have contributed to inconsistencies between BCS rates reported here and BCS rates in previous studies. The fact that we have been unable to show a significant benefit of tailored exemestane‐based neoadjuvant therapy in terms of increasing BCS rates (in this or our previously reported study^8^), despite the evidence of tumor regression, may be considered a factor against the use of this approach in postmenopausal patients with ER‐positive breast cancer. However, classification of patients into responders and nonresponders, as described in this article, results in similar clinical response rates in both groups and spares patients for whom treatment with exemestane alone is likely to be successful from the unpleasant effects of chemotherapy.

We believe that the present study is the first to investigate the efficacy and safety of combination therapy with exemestane plus TC in patients with an inadequate response to an initial period of treatment with exemestane alone. In conducting this study, we followed the recommendations of the Breast International Group and North American Breast Cancer Group Biomarker Working Party for the immunohistochemical assessment of Ki67 labeling index levels and the interpretation of Ki67 labeling index values.^26^ For example, in the absence of consensus regarding a recommended cutpoint, we considered a conservative cutpoint of 5%, combined with clinical response to the initial treatment with exemestane alone, appropriate for the study population. This cutpoint was based on the results of one of our previous studies, in which breast cancer patients with pPR had Ki67 labeling index ≤5% after 24 weeks' neoadjuvant exemestane therapy.^7^

The limitations of the present study are as follows. First, the study population was small (58 eligible patients), which limits the generalizability of our findings to larger populations. However, they add to those of previous studies, including those conducted by other research groups,^10^, ^29^ to suggest that Ki67 values after short‐term neoadjuvant endocrine therapy for primary breast cancer may have prognostic value. Second, interobserver variability in Ki67 labeling measurement cannot be ruled out. However, measurements may be expected to be more consistent at the low cutpoint of 5% used in our study than in the midrange of 8%‐15%,^33^ and the tailoring of treatment was not based solely on Ki67 labeling index. Third, in patients who were switched to exemestane plus TC, about one in three discontinued the study and one in five of the remainder did not comply with treatment; this would have affected outcomes in this group. Finally, PEPI scores were originally derived from the pT stage, pN stage, Ki67 labeling index level, and ER status of the surgical specimen after the initial treatment with exemestane alone, and not after treatment with exemestane plus TC.

In postmenopausal patients with ER‐positive breast cancer, tailored treatment offers the potential to maximize the effectiveness of neoadjuvant therapy and minimize the incidence of chemotherapeutic toxicity.^6^ Therefore, we await with interest the findings of ongoing trials in which the clinical utility of Ki67 labeling index is being investigated. These include the ALTERNATE trial (clinical trial no. NCT01953588, estimated date of completion, April 2020), which has been designed to assess a treatment strategy based on Ki67 values during neoadjuvant endocrine therapy in a similar study population to that of the present study.^34^

## 5 CONCLUSIONS

The results of our present study add to those of our previous studies in providing support for the use of tailored approaches in which criteria based on both pathological and clinical characteristics are used to identify patients for whom a switch from endocrine therapy to chemoendocrine therapy would maximize therapeutic effects while minimizing the incidence of AEs associated with chemotherapeutic drugs. They also confirm the utility of Ki67 labeling index as a potential predictive marker for guiding clinical decision making during exemestane‐based neoadjuvant therapy for postmenopausal patients with ER‐positive breast cancer.

## CONFLICTS OF INTEREST

NS has received remuneration from Chugai Pharmaceutical, Eisai, Pfizer, and Sysmex. NM has received remuneration from Chugai Pharmaceutical, AstraZeneca, Pfizer, Eli Lilly, Eisai and Takeda Pharmaceutical, and research funding from Chugai, AstraZeneca, Kyowa Hakko Kirin, MSD, Novartis Pharma, Pfizer, Eli Lilly, Eisai and Daiichi Sankyo. TU has received remuneration from Chugai Pharmaceutical, Eisai, and Novartis Pharma. SS has received remuneration from Chugai Pharmaceutical, Novartis Pharma, Kyowa Hakko Kirin, Esai, Takeda Pharmaceutical, and Pfizer, and research funding from Chugai Pharmaceutical and AstraZeneca. SM has received remuneration from Pfizer and Yakult. SO has received remuneration from Chugai Pharmaceutical, AstraZeneca, Pfizer, Kyowa Hakko Kirin, and Eizai. MT has received remuneration from Novartis Pharma, Takeda Pharmaceutical, AstraZeneca, Taiho Pharmaceutical, Chugai Pharmaceutical, Pfizer, Eisai, Eli Lilly, Kyowa Hakko Kirin, and Genomic Health, has a consultant/advisory role at Genomic Health, and has received research funding from Taiho Pharmaceutical and Chugai Pharmaceutical. TM, CK, KK, HY, and HS have nothing to disclose.

## Data Availability

The data that support the findings of this study are available on request from the corresponding author. The data are not publicly available due to privacy or ethical restrictions.
